# Supplementary material for: COZOID: contact zone identifier for visual analysis of protein-protein interactions
Source: BMC Bioinformatics. 2018 Apr 6;19:125. doi: 10.1186/s12859-018-2113-6 (PMC5889581; doi:10.1186/s12859-018-2113-6)
Supplement: Supplementary file 2 — Software build. Executable binary file of the software tool. (ZIP 118,784 kb) [file 12859_2018_2113_MOESM2_ESM.zip › cozoid/java/docs/beansbinding-1.2.1-doc.zip!/org/jdesktop/el/ELContextListener.html]

ELContextListener (Beans Binding)


---


|  |  |  |
| --- | --- | --- |
| **Overview** \| **Package** \| **Class** \| **Use** \| **Tree** \| **Deprecated** \| **Index** \| **Help** | |  |
| **PREV CLASS**   **NEXT CLASS** | **FRAMES**    **NO FRAMES**     **All Classes** |  |
| SUMMARY: NESTED \| FIELD \| CONSTR \| METHOD | DETAIL: FIELD \| CONSTR \| METHOD |  |


---


## org.jdesktop.el Interface ELContextListener

**All Superinterfaces:**: java.util.EventListener

---

``` public interface ELContextListener extends java.util.EventListener ```

The listener interface for receiving notification when an
`ELContext` is created.

**Since:**
:   JSP 2.1

**See Also:**: `ELContext`, `ELContextEvent`

---

| **Method Summary** | |
| --- | --- |
| `void` | `contextCreated(ELContextEvent ece)`             Invoked when a new `ELContext` has been created. |

**Method Detail**

### contextCreated

```
void contextCreated(ELContextEvent ece)
```

:   Invoked when a new `ELContext` has been created.

    :   **Parameters:**: `ece` - the notification event.


---


|  |  |  |
| --- | --- | --- |
| **Overview** \| **Package** \| **Class** \| **Use** \| **Tree** \| **Deprecated** \| **Index** \| **Help** | |  |
| **PREV CLASS**   **NEXT CLASS** | **FRAMES**    **NO FRAMES**     **All Classes** |  |
| SUMMARY: NESTED \| FIELD \| CONSTR \| METHOD | DETAIL: FIELD \| CONSTR \| METHOD |  |


---
